# Correlation between COVID-19 severity and previous exposure of patients to *Borrelia* spp.

**DOI:** 10.1038/s41598-022-20202-x

**Published:** 2022-09-24

**Authors:** Alina Szewczyk-Dąbrowska, Wiktoria Budziar, Marek Harhala, Krzysztof Baniecki, Aleksandra Pikies, Natalia Jędruchniewicz, Zuzanna Kaźmierczak, Katarzyna Gembara, Tomasz Klimek, Wojciech Witkiewicz, Artur Nahorecki, Kamil Barczyk, Marlena Kłak, Urszula Grata-Borkowska, Krystyna Dąbrowska

**Affiliations:** 1 Regional Specialist Hospital in Wrocław, Research and Development Center, Kamieńskiego 73a, Wrocław, Poland; 2 Department of Family Medicine, Wroclaw Medical University, 1 Syrokomli St, 51-141 Wroclaw, Poland; 3 Healthcare Centre in Bolesławiec, Jeleniogórska 4, Bolesławiec, Poland; 4 Hirszfeld Institute of Immunology and Experimental Therapy, Weigla 12, Wrocław, Poland

**Keywords:** Bacteria, Virology

## Abstract

Predictors for the risk of severe COVID-19 are crucial for patient care and control of the disease. Other infectious diseases as potential comorbidities in SARS-CoV-2 infection are still poorly understood. Here we identify association between the course of COVID-19 and Lyme disease (borreliosis), caused by *Borrelia burgdorferi* transmitted to humans by ticks. Exposure to *Borrelia* was identified by multi-antigenic (19 antigens) serological testing of patients: severe COVID-19 (hospitalized), asymptomatic to mild COVID-19 (home treated or not aware of being infected), and not infected with SARS-CoV-2. Increased levels of *Borrelia*-specific IgGs strongly correlated with COVID-19 severity and risk of hospitalization. This suggests that a history of tick bites and related infections may contribute to the risks in COVID-19. Though mechanisms of this link is not clear yet, screening for antibodies targeting *Borrelia* may help accurately assess the odds of hospitalization for SARS-CoV-2 infected patients, supporting efforts for efficient control of COVID-19.

## Introduction

Borreliosis, also known as Lyme disease, is a zoonosis caused by the bacterium *Borrelia burgdorferi* transmitted to humans by ticks. A characteristic symptom of infection is the skin rash called erythema migrans, which however occurs in only 60–80% of infected persons, with a delay of 3–30 days after the tick bite. Other typical symptoms are non-specific, including fever, mild headaches, muscular-articular pain, and fatigue. Thus, a considerable number of cases of *Borrelia* infection can be overlooked, while if left untreated, infection can spread to joints, the heart, and the nervous system^1,2^. Subjective symptoms may persist or recur for at least six months after a patient was infected, even after antimicrobial treatment^3,4^. So-called ‘chronic Lyme disease’, sometimes with severe manifestations, remains controversial, lacking clearly recognized or demonstrated significance for a patient’s health^5^.

The diagnostics of *Borrelia* infection is challenging, primarily due to the multitude of bacterial strains that may cause the infection. In Eurasia, *Borrelia burgdorferi* sensu lato includes various spirochete species: *B. burgdorferi* sensu stricto (s.s.), *B. afzelii*, *B. garinii*, *B. bavariensis*, *B. japonica*, *B. lusitaniae*, *B. sinica*, *B. spielmanii*, *B. tanukii*, *B. turdi*, *B. valaisiana*, *B. yangtze*, *B. bissettii*, and *B. carolinensis*. Within the area of this study (Central Europe), the predominant species as the causative agents of borreliosis are *Borrelia afzelii* and *Borrelia garinii*, less frequently *Borrelia burgdorferi* s.s.^6–8^ *. B. spielmanii* has also been found in an animal reservoir, postulated as an appropriate antigenic component in diagnostic tests in Europe^8,9^.

Serological testing of patients’ sera with antigens representing pathogenic species of *Borrelia* is the major approach in diagnostics^10^, even though seropositivity alone is not sufficient for a diagnosis of active borreliosis, since antibodies may persist for long after treatment of the disease and they can be detected even in individuals after a successful treatment ^3,4^. Proper interpretation of a patient’s clinical status is difficult due to different life strategies of *B. burgdorferi*; these are related to their antigenic variability, which is in turn linked to adaptation to different environmental conditions, their intracellular residence, and their hiding in immunologically privileged areas^11,12^. Presence of antibodies targeting multiple antigens of Borrelia, however, indicates that an individual has been infected, without a clear indication of whether the infection has been eradicated or it is still active. Thus, serological testing remains the major tool available for epidemiological studies^10^.

The emergence of COVID-19, a new disease caused by the previously unknown SARS-CoV-2 virus, is linked to still very high uncertainty about factors that determine the clinical manifestation and course of this disease. The course of COVID-19 ranges from asymptomatic or mild to severe or fatal with fulminant development and dramatic symptoms. Some crucial conditions in COVID-19 have been revealed, including comorbidities such as diabetes, cardiovascular diseases, cancer, pulmonary disorders, and immunological disorders, but also obesity, advanced age, and others^13–15^. Infectious diseases, however, have been much less recognized as comorbidities in SARS-CoV-2 infection and their potential effect on the risk of severe COVID-19 is poorly understood. Particularly, the possible association between Lyme disease and COVID-19 disease has not been established, in spite of indications that flu-like symptoms reported in both Lyme disease and COVID-19 can be very similar^16^. One available case report by Shutikova et al. (2021) describes a patient with disseminated *Borrelia* infection (starting in mid-2019), after an unsuccessful first round of antibiotic treatment (early 2020), who was infected with SARS-CoV-2 (mid-2020). Anti-COVID-19 treatment included umifenovir, hydroxychloroquine, azithromycin, and ceftriaxone, and it resulted in suppression of borreliosis, as manifested by the first decrease of anti-*Borrelia* IgG in diagnostics. However, that report was not dedicated to possible effects of borreliosis on the course of COVID-19^17^. Piotrowski and Rymaszewska (2022) pointed out a reduced number of cases of Lyme disease registered during the COVID-19 pandemic in Poland, and they concluded that it may result from limited outdoor activities in the lockdown, but possibly also from poor access of patients to the overburdened health care system^18^.

Further studies, for instance on the potential association of borreliosis with increased risk of SARS-CoV-2 infection or with severe COVID-19, have not been reported so far. In this study we analyzed a wide range of anti-Borrelia IgG and IgM antibodies (targeting 19 antigens derived from *Borrelia* spp.) in patients representing three different types of clinical history: severe COVID-19 (hospitalized), asymptomatic to mild COVID-19 (home treated or not aware of being infected), and not infected with SARS-CoV-2, to identify potential association between previous exposure to *Borrelia* spp. and the risk of SARS-CoV-2 infection or severe COVID-19.

## Results

A multi-antigen Microblot-Array for the diagnostics of *Borrelia* species was conducted in three groups of patients: with severe COVID-19 (hospitalized), with asymptomatic to mild COVID-19 (home treated or not aware of being infected), and those not infected with SARS-CoV-2. Demographic parameters of the investigated population were different to general demographic parameters of the whole country population, due to the imbalanced representation of hospitalized COVID-19 patients, who tend to be elderly. The studied groups, however, were agreed for concordant demographic parameters without statistically significant differences (Supplementary Figs. S1 and S2). The testing revealed that all patients hospitalized due to COVID-19 disease were positive for *Borrelia burgdorferi*-specific IgG (31 out of 31). In patients with mild/asymptomatic COVID-19, 19 positive cases were found (out of 28), and in participants never infected with SARS-CoV-2 only 8 positive cases were found (out of 28) (as assessed according to the manufacturer’s instructions for array interpretation).

**Figure 1:**
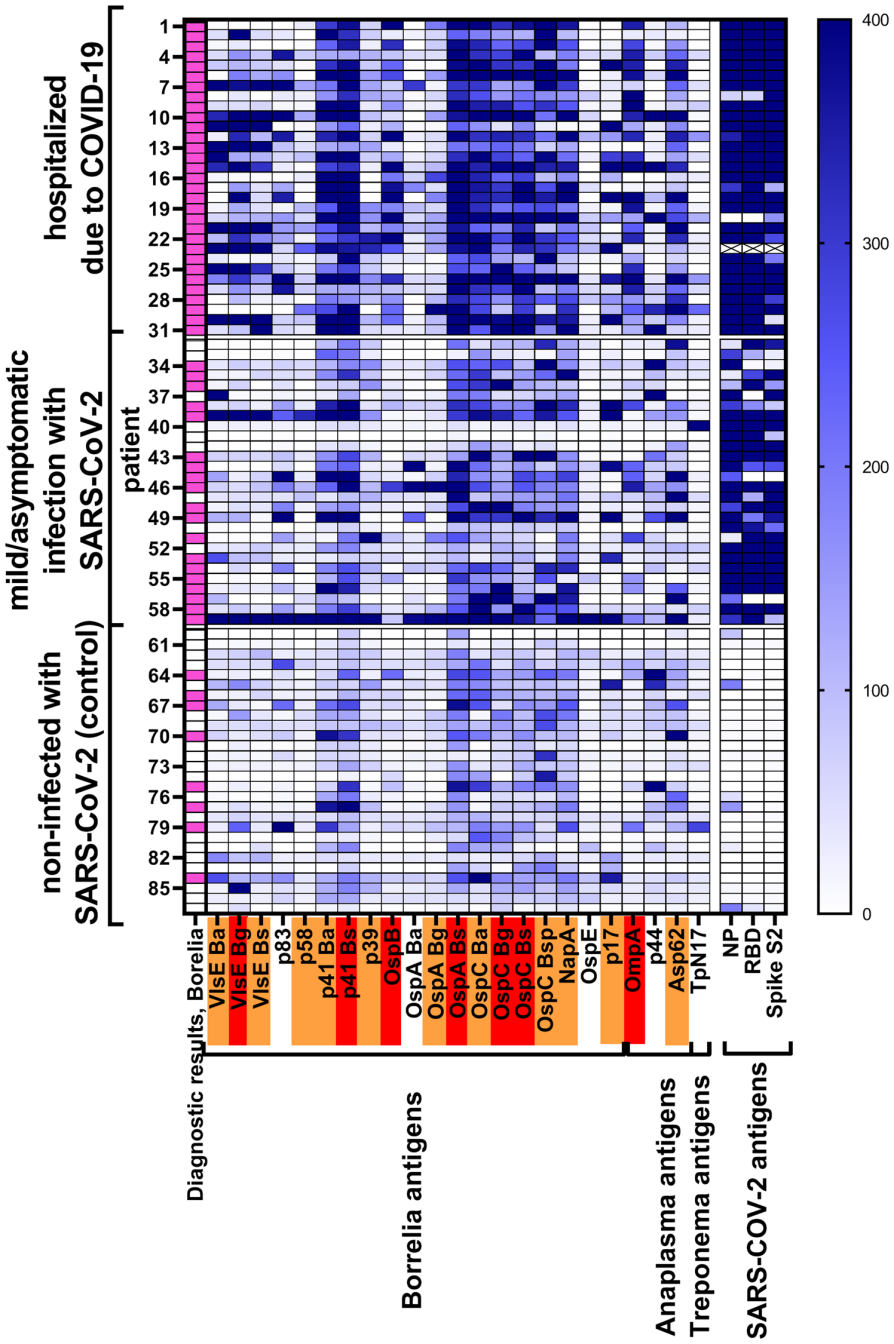
Serum levels of *Borrelia*-specific IgG in patients representing three different types of clinical history for COVID-19: severe (hospitalized), asymptomatic to mild (home treated or not aware of being infected), and not infected with SARS-CoV-2 (seronegative). Each row represents one sample from a patient (numbers from 1 to 87). Highlighted in red – antigens for which IgG reactivity was significantly different between hospitalized patients and two other groups, highlighted in orange – antigens for which IgG reactivity was significantly different between hospitalized patients and those not infected with SARS-CoV-2, diagnostic results- identification of positive (magenta) or negative (white) diagnosis for *Borrelia* IgG as assessed according to the manufacturer’s instructions for array interpretation. Description of all antigens is given in Table 1.

For 6 out of 19 *Borrelia* antigens tested, severe COVID-19 patients demonstrated significantly higher specific IgG serum levels than any other group of patients: VlsE *B. garinii*, p41 *B. burgdorferi* sensu stricto, OspB, OspA *B. burgdorferi* sensu stricto, OspC *B. garinii*, and OspC *B. burgdorferi* sensu stricto. For a further 10 *Borrelia* antigens, severe COVID-19 patients also demonstrated significantly higher specific IgG serum levels than participants not infected with SARS-CoV-2: VlsE *B. afzelii*, VlsE *B. burgdorferi* sensu stricto, p58, p41 *B. afzelii*, p39, OspA *B. garinii*, OspC *B. afzelii*, OspC *B. spielmanii*, Nap A and p17 (Fig. 1, all *p*-values are listed in Supplementary Table S1, numbers of patients testing positively for each antigen-specific IgG are listed in Supplementary Table S2). This observation suggests that previous exposure to *Borrelia* renders patients more prone to severe COVID-19 in case of SARS-CoV-2 infection.

Severe COVID-19 patients also demonstrated significantly higher levels of IgGs specific to *Anaplasma* antigens (OmpA and Asp62) (Fig. 1), while *Anaplasma* is often co-transmitted with *Borrelia* by ticks. This further supports the suggestion that increased risks in COVID-19 are linked to a history of ticks bites and related infections.

**Figure 2:**
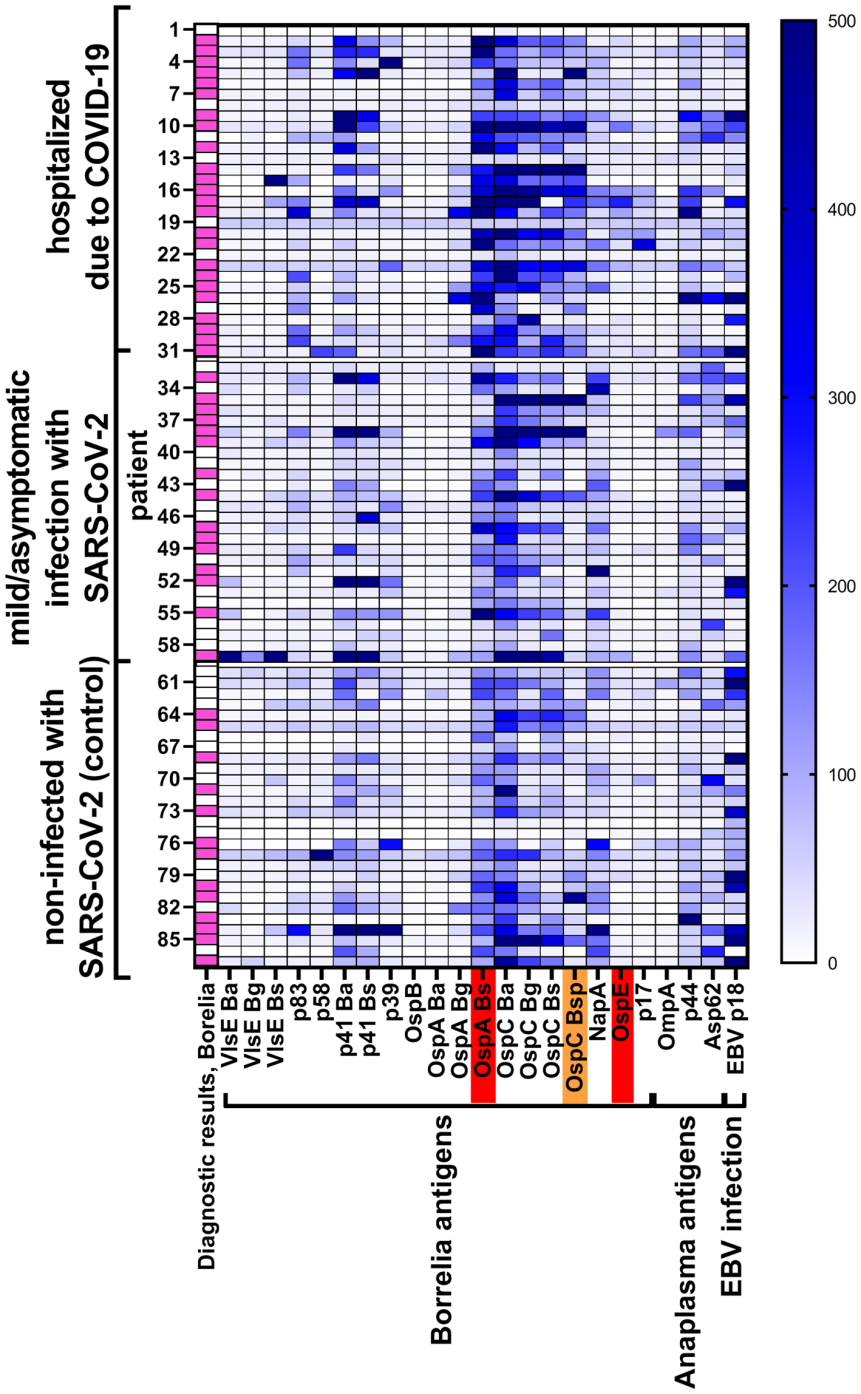
Serum levels of *Borrelia*-specific IgM in patients representing three different types of clinical history for COVID-19: severe (hospitalized), asymptomatic to mild (home treated or not aware of being infected), and not infected with SARS-CoV-2 (seronegative). Each row represents one sample from a patient (numbers from 1 to 87). Highlighted in red – antigens for which IgM reactivity was significantly different between hospitalized patients and two other groups, highlighted in orange – antigens for which IgM reactivity was significantly different between hospitalized patients and those not infected with SARS-CoV-2, diagnostic results- identification of positive (magenta) or negative (white) diagnosis for *Borrelia* IgG as assessed according to the manufacturer’s instructions for array interpretation. Description of all antigens is given in Table 1.

Testing for *Borrelia*-specific IgM revealed that among patients hospitalized due to COVID-19 disease, 24 (out of 31) were positive for *Borrelia burgdorferi*-specific IgM, while in mild/asymptomatic patients and in participants never infected with SARS-CoV-2 only 13 (out of 28) and 15 (out of 28) positive cases were found, respectively. Significant differences between groups of patients in their IgM reactivity with the tested antigens were found for OspA *B. burgdorferi* sensu stricto and OspE; for these antigens severe COVID-19 patients demonstrated significantly higher specific IgM levels than the two other groups of patients. Also for OspC *B. spielmanii*, severe COVID-19 patients demonstrated significantly higher specific IgM levels than the group of participants seronegative to SARS-CoV-2 (Fig. 2, all *p*-values are listed in Supplementary Table S3, numbers of patients testing positively for each antigen-specific IgM are listed in Supplementary Table S4).

**Figure 3:**
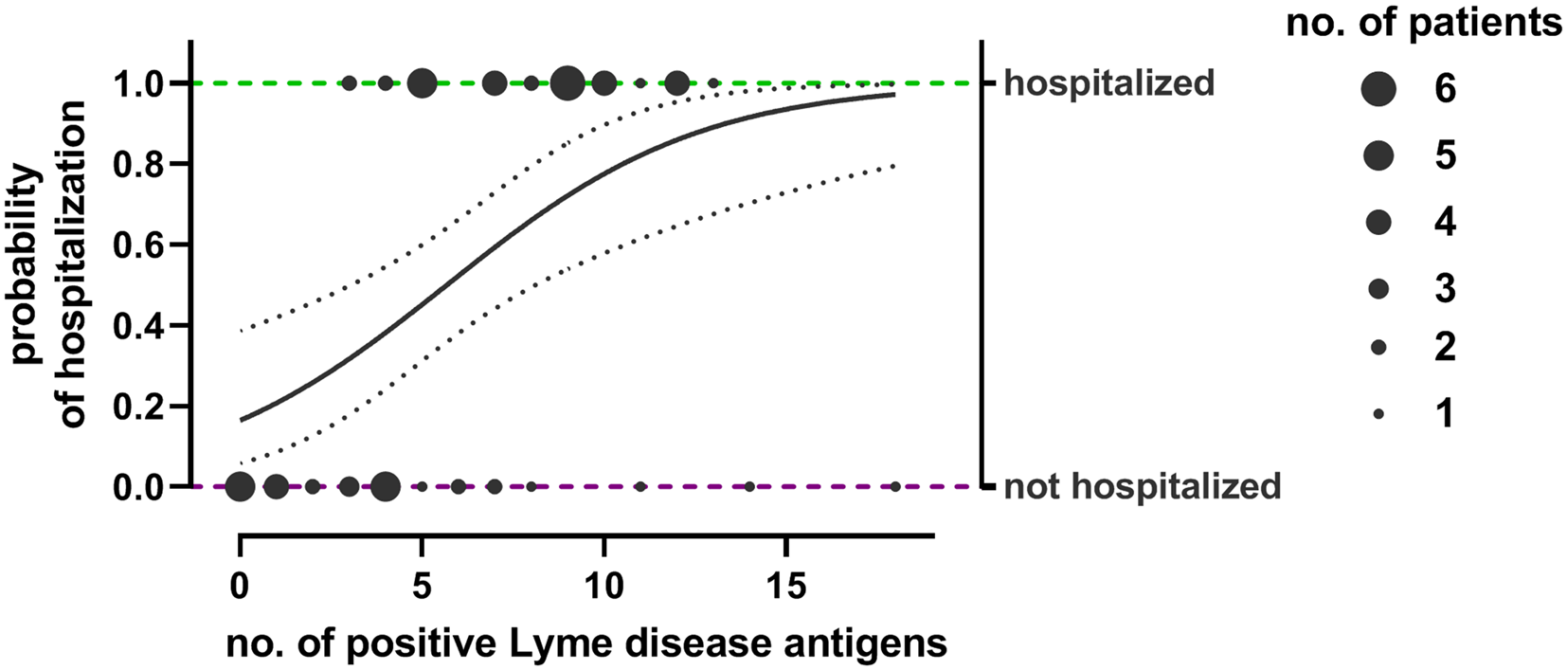
A model for association between number of *Borrelia* antigens recognized by patients’ IgG (predictor variable) and hospitalization due to COVID-19 infection (response variable). Size of dots represents number of patients hospitalized or not. Logistic regression (line) was applied (odds ratio = 1.47, *p* = 0.0002).

Logistic regression was used as a model for testing the association between number of *Borrelia* antigens recognized by patients’ IgG (predictor variable) and hospitalization due to COVID-19 infection (response variable). We found that the odds (probability) of hospitalization for COVID-19 increase with each positive read for a Lyme disease-related antigen for IgG antibodies (*p* < 0.05, Fig. 3) but not for IgM (Supplementary Fig. S3). Post-hoc analysis of selected antigens (Osp proteins, p41, and VlsE) was also included; multivariate analysis showed that odds of hospitalization increased with increasing levels of IgG antibodies targeting OspB, OspC *B. burgdorferi* sensu stricto, and OspC *B. spielmanii* (logistic regression, *p* < 0.05) (Supplementary Table S5. For IgM antibodies, the same association was observed for antibodies targeting OspC *B. spielmanii* and OspE (logistic regression, *p* < 0.05) (Supplementary Table S6).

## Discussion

In this study we investigated potential correlations between detected antibody levels indicating exposure to *Borrelia* and the risk of increased severity of COVID-19. Previous exposure to *Borrelia* was identified by multi-antigenic serological testing, and it revealed that increased levels of *Borrelia*-specific IgGs strongly correlated with COVID-19 severity and with the risk of hospitalization (Fig. 1 and 3, Supplementary Tables S1 and S2). For *Borrelia*-specific IgMs, correlations were weaker and mostly insignificant (Fig. 2 and Supplementary Fig. S3, Supplementary Tables S3 and S4).

**Figure 4:**
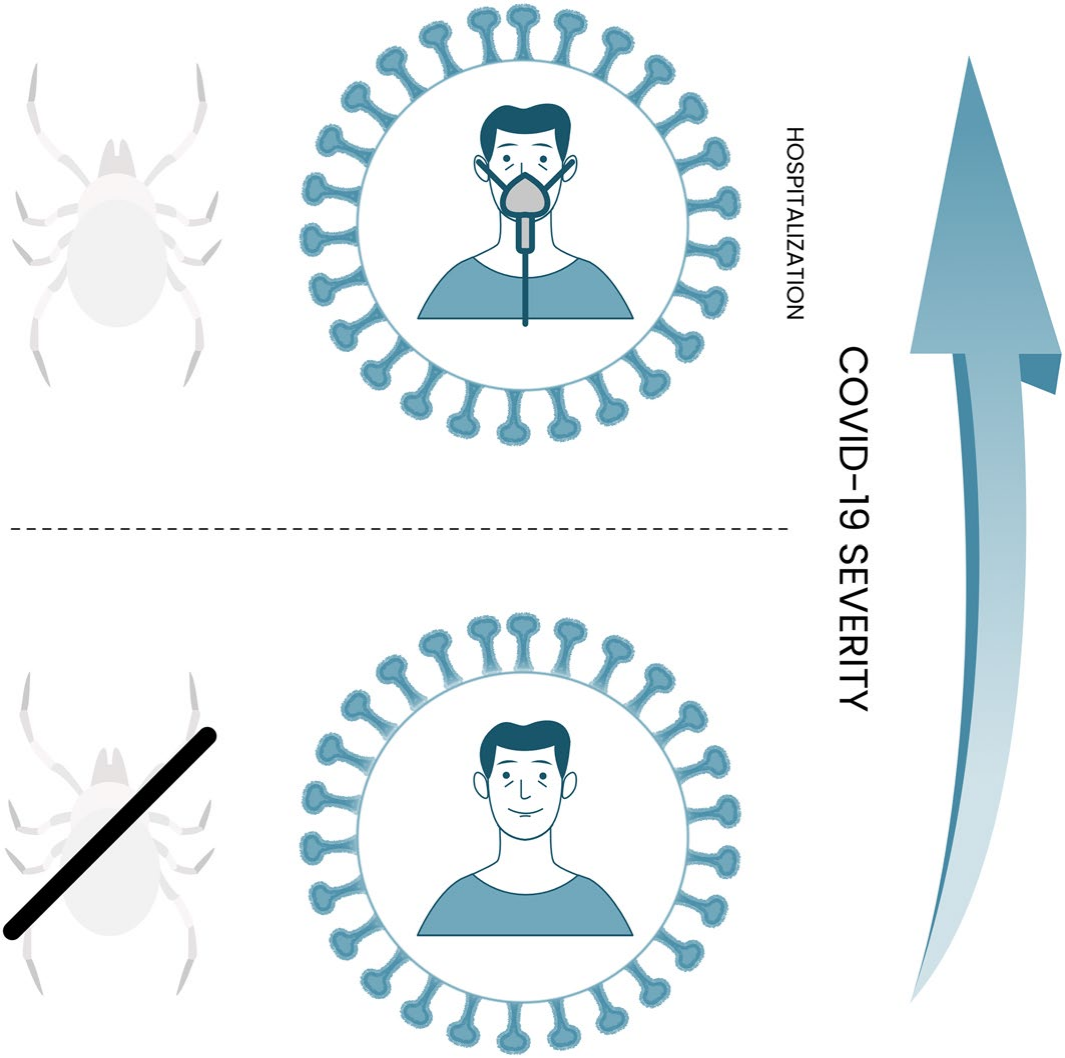
Risks in COVID-19 are linked to a history of tick bites and related infections.

Typically, pathogen-specific IgM increases at the early stage of infection, while IgG development takes more time. In borreliosis, at the early stage of infection (2–4 weeks) the immunological system detects only a few antigens of *Borrelia*, e.g. p41 (flagellin) and Osp proteins (outer surface proteins), targeted by IgM antibodies. *Borrelia*-specific IgGs, in turn, can be observed several weeks after the tick bite, and their increased serum concentration can remain for a long time, even after the resolution of clinical symptoms. OspC, OspA, and p41 are considered the most immunogenic proteins of *B. burgdorferi*^19–21^; consistently, in this study IgGs targeting these antigens were also the most frequent and they reached the highest levels (Fig. 1). Other important targets for IgG diagnostics include VisE, p83, p58, and p17 ^19–21^, also detected in this study. Interestingly, in many patients we observed antibodies targeting different species (e.g. *B. burgdorferi* sensu stricto, and at the same time *B. afzelii*, and/or *B. garinii*). This may reflect some cross-reactivity of antibodies, but likely it may result from co-infections with more than one species, which according to the literature may also occur ^22^. Also, severe COVID-19 patients demonstrated significantly higher levels of IgG specific to *Anaplasma* (Fig. 1), which is often co-transmitted with *Borrelia* by ticks. This further supports the suggestion that increased risks in COVID-19 are linked to a history of tick bites and related infections (Fig. 4).

Important limitations should be considered for a full understanding of this study’s results. First, diagnostics of Lyme disease (active borreliosis) is still difficult and often unclear. Laboratory testing should be considered in conjunction with potential exposure and compatible clinical symptoms^10^; data on patients’ history of tick bites and on potential borreliosis-related symptoms were not available here. Particularly severe COVID-19 patients under intensive care were not able to give them. Thus, in the investigated group at least some individuals may demonstrate immunological memory of previous *Borrelia* infection/s but not an active disease. On the other hand, difficulties with rapid and unambiguous diagnosis may lead to some *Borrelia*-infected patients going untreated, with the pathogen affecting their health condition even for a long time.

Second, although this study demonstrated a significant correlation between serum levels of anti-*Borrelia* antibodies and COVID-19 severity observed in the same individuals, a correlation cannot be assumed to indicate causation. One cannot exclude that there was an unidentified primary factor that in these patients caused both higher vulnerability to *Borrelia* infection and to severe COVID-19. This could possibly be immunodeficiency, other physiological disorders or comorbidities. Of note, patients in the severe COVID cohort were likely to have more comorbidities than those in the other two groups. For instance, obesity has been indicated as associated with the risk of COVID-19-related hospitalizations and death^23^. In Lyme disease, obesity was associated with attenuated and delayed IgG responses to *B. burgdorferi*, thus suggesting less efficient protection from adaptive immunity in obese individuals^24^. Since these patients demonstrated an efficient antibody response to SARS-CoV-2 (Fig. 1), this issue calls for further research. Demographic parameters, in turn, have been agreed between groups (Supplementary Fig. S2), so for instance elderly age was not a contributing factor here.

Alternatively, prolonged Lyme disease might affect the immune system, decreasing its efficacy in antiviral responses in the viral infection. This has never been demonstrated yet, though important effects that *Borrelia* may have on the immune system have been described^25,26^. Furthermore, one of the possible explanations for studied relationship may be a more detailed insight into the mechanisms of the immune system, more specifically the Toll-like receptor pathway (TLR), whose innate immunity receptors recognize ligands derived from bacteria, fungi, and viruses^27^. Studies indicate that the TLR pathway mediates, at least in part, the release of inflammatory mediators in human monocytes stimulated with live *B. burgdorferi* spirochetes^28^. Similarly, the role of TLR receptors has been described in SARS-CoV-2 infection, which contributes to the elimination of viruses, but it can also harm the host due to persistent inflammation and tissue destruction^29^. Particularly, *B. burgdorferi* has been demonstrated to interact with TLR1/TLR2 heterodimers with resulting stimulation of inflammatory response, including increased inflammatory cytokine markers, like IL-6 and TNF-α^28^. The same molecular pathway is targeted by SARS-CoV-2, where stimulation via TLR1 and TLR2 has been indicated as the key factor of excessively upregulated cytokine response and its harmful effects within severe COVID-19^30,31^. This suggests that co-stimulation from both *B. burgdorferi* and SARS-CoV-2 may result in even more pronounced excessive inflammatory response and a higher risk of severe COVID-19. This hypothesis needs to be further verified in future studies.

In spite of above mentioned important reservations and considerations, a strong link between detected anti-*Borrelia* antibodies and COVID-19 severity was observed in this study (Fig. 1, 2, and 3). This was further supported by post-hoc analysis of IgG targeting selected antigens of *Borrelia*. These antigens included Osp proteins, p41, and VlsE, being highly immunogenic^19–21^ and important in the life cycle of spirochetes; they are engaged in bacterial colonization of ticks and mammals, virulence, and immune evasion by *Borrelia*^32–34^. The analysis with multivariant logistic regression revealed that increased levels of IgG targeting Osp proteins (only) can be significant predictors of hospitalization due to COVID-19; in this study OspB, OspC *B. burgdorferi* sensu stricto, and OspC *B. spielmanii* demonstrated significance in this model (Supplementary Fig. S4, Supplementary Table S5).

To the best of our knowledge, this is the first observation that suggests links between Lyme disease and COVID-19 prognostics. Screening for antibodies targeting *Borrelia* may contribute to accurately assessing the odds of hospitalization for SARS-CoV-2 infected patients. Though mechanisms of this association are not clear yet, it may help in establishing optimal treatment schedules and in efficient predictions of individual patients’ prognostics, supporting efforts for efficient control of COVID-19.

## Methods

### Participants

*Group 1 (hospitalized)* Participants with severe COVID-19: infection with SARS-CoV-2 confirmed with PCR diagnostics, hospitalized due to their poor condition (Healthcare Centre in Bolesławiec); these patients required either i) transient, non-invasive assisted respiratory therapy, ii) assisted respiratory therapy with the nasal high-flow cannula, or iii) invasive mechanical ventilation (*N* = 31);

*Group 2 (asymptomatic/mild)* Participants with mild or asymptomatic COVID-19, who were treated at home or unaware of being infected, non-vaccinated against COVID-19 but identified as seropositive to anti-SARS-CoV-2 antibodies ^35^ (*N* = 28);

*Group 3 (control)* Participants without diagnosis and history of COVID-19 disease, non-vaccinated against COVID-19 and identified as seronegative to anti-SARS-CoV-2 antibodies^35^ (*N* = 28). The studied groups were agreed for concordant demographic parameters.

### Blood samples

Serum was collected into tubes (BD SST II Advance), left for 1 h at room temperature (RT) to clot and separated from the clot by centrifugation (15 min, 2000 g, RT), and then stored at –20 °C for further use.

### Bioethics statements

The research was approved by the local Bioethical Commission of the Regional Specialist Hospital in Wroclaw (approval no. KB/02/2020, policy no. COR193657) and the procedures were in line with the Declaration of Helsinki. During the interview, all information about the study was provided and written informed consent was obtained from each study participant.

### Serological diagnostic tests

Serological identification of SARS-SoV-2 infection was conducted with: Microblot—Array COVID-19 IgG (TestLine Clinical Diagnostics s.r.o.); the array contains selected fragments of specific NCP, RBD and Spike S2 antigens of SARS-CoV-2 virus. According to the manufacturer’s information, this IgG-detecting microblot-array demonstrates diagnostic sensitivity 98.7% and diagnostic specificity 99.3%. The tests have been standardized against the 1st WHO international standard for anti-SARS-CoV-2 immunoglobulin (human): NIBSC code: 20/136 and regulatory status of this test in European Union is IVD CE (the device complies with the European In-Vitro Diagnostic Devices Directive (IVDD 98/79/EC).

Serological identification of *Borrelia* spp. infection was conducted with: Microblot-Array Borrelia IgG and Microblot-Array Borrelia IgM (TestLine Clinical Diagnostics s.r.o.); the arrays contain specific antigens of *Borrelia* spp.: VlsE *B. afzelii*, VlsE *B. garinii*, VlsE *B. burgdorferi* sensu stricto, p83, p58, p41 *B. afzelii*, p41 *B. burgdorferi* sensu stricto, p39, OspB, OspA *B. afzelii*, OspA *B. garinii*, OspA *B. burgdorferi* sensu stricto, OspC *B. afzelii*, OspC *B. garinii*, OspC *B. burgdorferi* sensu stricto, OspC *B. spielmanii*, OspE, NapA, and p17. They also include antigens specific for *Anaplasma phagocytophilum* since this pathogen is often co-transmitted from ticks to a host together with *Borrelia* spp. (p44, OmpA and Asp62), recombinant antigen TpN17 (IgG) and EBV p18 (IgM) to exclude false-positive reads that might result from cross-reactivity to *Treponema pallidum* or Epstein-Barr virus. A description of all investigated antigens is presented in Table 1. According to the manufacturer’s information, IgG-detecting microblot-array demonstrates diagnostic sensitivity: 97.3% for Borrelia, 92.0% for Anaplasma, 98.3% for Treponema, and diagnostic specificity 98.0% for Borrelia, 100.0% for Anaplasma, 100.0% for Treponema. IgM-detecting microblot-array demonstrates diagnostic sensitivity: 94.6% for Borrelia, 95.0% for Anaplasma, 100.0% for EBV, and diagnostic specificity 95.8% for Borrelia, 100.0% for Anaplasma, 98.0% for EBV. Regulatory status of these tests in European Union is IVD CE (the device complies with the European In-Vitro Diagnostic Devices Directive (IVDD 98/79/EC).

**Table 1:** Antigens tested in this study; Ba – *B. afzelii*, Bg – *B. garinii*, Bs – *B. burgdorferi* sensu stricto, Bsp – *B. spielmanii* (modified from manufacturer’s instructions: BioVendor Group, TestLine Clinical Diagnostics, Brno, Czech Republic, Microblot-Array Manual).

Antigen	Description
VlsE Ba	Expressed part of variable major protein-like sequence, significant for IgG antibody response, species-specific antigen
VlsE Bg	
VlsE Bs	
A4 p83	Main extracellular protein (product of p100 degradation)
p58	OppA-2 (oligopeptide permease 2) – membrane transporter, is considered a marker of disseminated stage of Lyme disease
p41 Ba	Internal flagellin, highly specific antigen of early antibody response
p41 Bs	
p39	BmpA (glycosaminopeptide receptor) – marker of late IgG antibody response
OspB	Outer surface protein B, marker of late stage of infection, considered a marker of Lyme arthritis
OspA Ba	Outer surface protein A, highly specific marker of *Borrelia* infection in IgG class
OspA Bg	
OspA Bs	
OspC Ba	Outer surface protein C – main antigen of early antibody response, immunodominant marker of IgM antibody response
OspC Bg	
OspC Bs	
OspC Bsp	
OspE	Outer surface protein E
NapA	Neutrophil activating protein A – strong immunogen, main marker of Lyme arthritis pathogenesis
p17	DbpA (decorin-binding protein A) – outer membrane protein
p44	Anaplasma phagocytophilum – main marker of human granulocytic anaplasmosis antibody response
OmpA	Outer membrane protein A of *Anaplasma phagocytophilum*; peptidoglycan-associated lipoprotein, significant virulence marker
Asp62	Surface protein – membrane transporter
TpN17	Highly specific membrane protein of *Treponema pallidum*
VCA-p18	Viral capsid antigen p18 – important marker of EBV infection

### Statistical methods

Statistical significance of differences between groups in serum levels of *Borrelia* spp. antibodies (factor with 3 levels) was tested with one-way ANOVA with the assumption of non-equal SD and Gaussian distribution (Brown-Forsythe and Welch ANOVA test). Dunnett’s T3 multiple comparisons test was used to determine the significance of differences between each pair of groups (GraphPad 9).

Logistic regression analysis was used to assess the risk of severe COVID-19 disease (hospitalization, outcome variable) depending on the presence or levels of antibodies against *Borrelia* antigens (predictor variable, main effects and intercept only). Spearman's test was used to calculate correlations between levels of antibodies. Statistical analyses were performed using GraphPad Prism 9 (version 9.1.3 for Windows, GraphPad Software, San Diego, California USA, www.graphpad.com). Graphs representing boxplots were plotted with the middle line representing the median; upper and lower border of a boxplot are the 75th and 25th percentile respectively. Whiskers represent the maximum and minimum (max/min) read (Tukey method, as plotted by GraphPad 9). Dots represent reads outside of the max/min range (outliers). Statistical tests were two-tailed.

## Supplementary Information

Supplementary Information 1.

Supplementary Information 2.

## Data Availability

All data generated or analysed during this study are included in this published article [and its supplementary information files].
