# Supplementary material for: Correlation between COVID-19 severity and previous exposure of patients to Borrelia spp
Source: Sci Rep. 2022 Sep 24;12:15944. doi: 10.1038/s41598-022-20202-x (PMC9509370; doi:10.1038/s41598-022-20202-x)
Supplement: Supplementary file 2 — Supplementary Information 2. [file 41598_2022_20202_MOESM2_ESM.pdf]

Supplementary

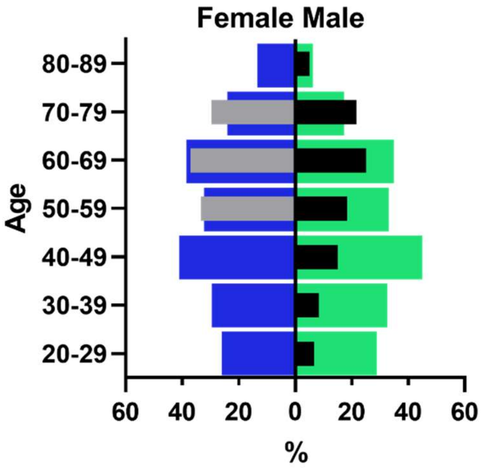

demographic parameters of all participants

demographic parameters of the Polish population (general)

Supplementary Fig. S1. Demographics of all study participants compared to Polish population; overrepresentation of elderly participants in the investigated groups was statistically significant ($p<0.0001$).

<table>
<tr><td>Group 1

(hospitalized)</td><td>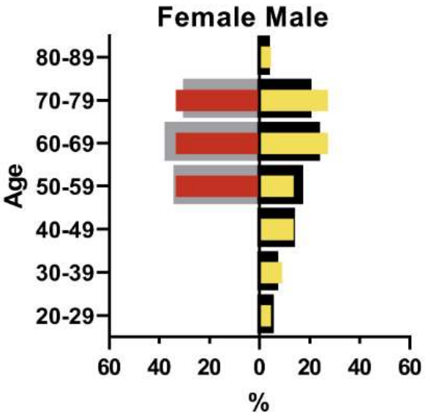
</td></tr>
<tr><td>Group 2

(asymptomatic

/mild)</td><td>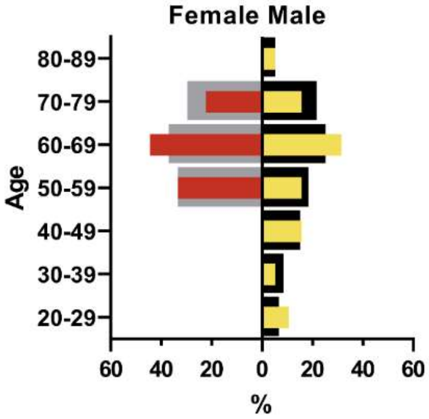
</td></tr>
<tr><td>Group 3

(control)</td><td>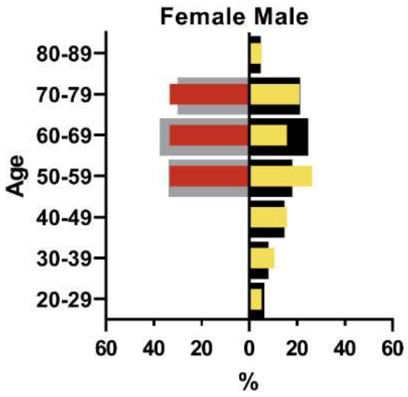
</td></tr>
<tr><td></td><td>demographic parameters of the study gropus
demographic parameters of all participants</td></tr>
</table>

Supplementary Fig. S2. Demographics of each study group compared to general demographics of all participants; no statistical significance was found (hospitalized due to COVID-19: p=0.9925; infected with SARS-CoV-2, not hospitalized: p=0.9183; without SARS-CoV-2 infection: p=0.9218)

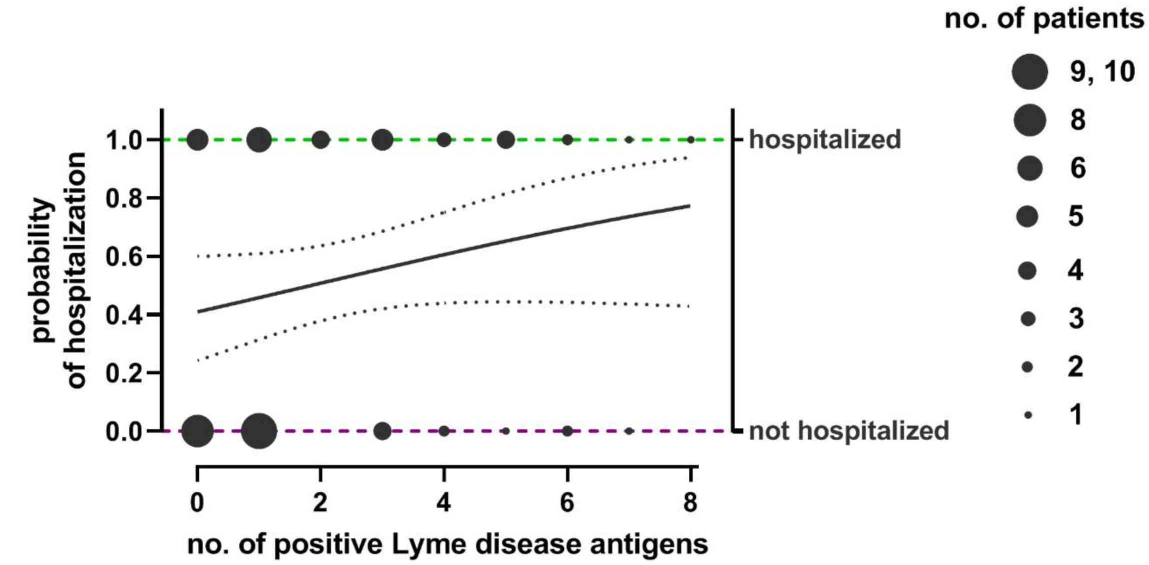

Supplementary Fig. S3. A model for association between number of *Borrelia* antigens recognized by patients' IgM (predictor variable) and hospitalization due to COVID-19 infection (response variable). Size of dots represents number of patients hospitalized or not. Logistic regression (line) was applied and found insignificant (p=0.117).

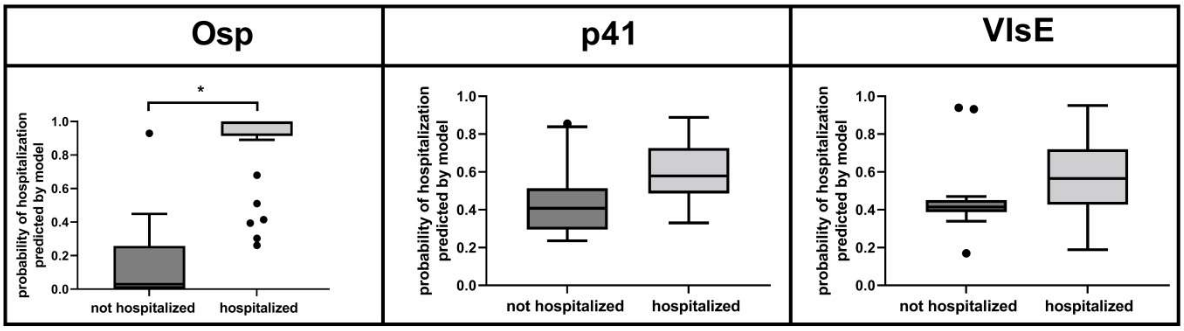

Supplementary Fig. S4. Linear regression model for probability of hospitalization (response variable) depending on serum levels of IgGs targeting selected *Borrelia* antigens (predictor variable): Osp proteins, or p41 (flagellin) proteins, or VlsE proteins; * p<0.05.

Supplementary Table S1. Statistical analysis of *Borrelia*-specific IgG levels in individuals hospitalized due to COVID-19 (Group 1), individuals with mild/asymptomatic SARS-CoV-2 infection (Group 2), and individuals not infected with SARS-CoV-2 (Group 3). Dunnett's T3 multiple comparisons tests were applied; p-values are presented. Antibodies were identified in patients' serum samples with *Borrelia*-dedicated Microblot-Array that quantified IgG levels (U/ml).

**Tested antigens**				**p-values**		
		**Welch's ANOVA**	**W(DFn, DFd)**	**Group 1 v. Group 2**	**Group 1 v. Group 3**	**Group 2 v. Group 3**
Borrelia	VlsE Ba	**0.0029**	6.764 (2.000,41.14)	0.4425	**0.006**	0.2591
	VlsE Bg	**0.0002**	9.942 (2.000, 47.38)	**0.027**	**0.0002**	0.8256
	VlsE Bs	**0.0008**	8.548 (2.000, 39.21)	0.1486	**0.0011**	0.4890
	p83	0.1875	1.726 (2.000, 55.14)	0.8606	0.2137	0.6063
	p58	**0.0064**	5.731 (2.000, 41.26)	0.9896	**0.0106**	0.3863
	p41 Ba	**<0.0001**	19.47 (2.000, 47.90)	0.0788	**<0.0001**	0.0661
	p41 Bs	**<0.0001**	27.27 (2.000, 49.43)	**0.0206**	**<0.0001**	**0.0257**
	p39	**0,0043**	6.146 (2.000, 45.36)	0.8699	**0.009**	0.2083
	OspB	**<0.0001**	18.91 (2.000, 51.58)	**<0.0001**	**<0.0001**	0.9990
	OspA Ba	**0.0654**	2.911 (2.000,42.29)	0.4743	0.4013	0.1458
	OspA Bg	**0.0024**	7.006 (2.000, 41.51)	0.8970	**0.0041**	0.3110
	OspA Bs	**<0.0001**	36.01 (2.000, 51.69)	**0.0004**	**<0.0001**	**0.0403**
	OspC Ba	**<0.0001**	31.07 (2.000, 52.76)	0.0725	**<0.0001**	**0.0216**
	OspC Bg	**<0.0001**	26.72 (2.000, 44.91)	**0.0413**	**<0.0001**	**0.0073**
	OspC Bs	**<0.0001**	33.60 (2.000, 45.64)	**<0.0001**	**<0.0001**	**0.0457**
	OspC Bsp	**<0.0001**	11.71 (2.000, 48.69)	0.1726	**0.0001**	0.0993
	NapA	**<0.0001**	18.89 (2.000, 51.05)	0.1159	**<0.0001**	**0.0046**
	OspE	**0.6355**	0.4575 (2.000, 50.02)	0.9517	0.8501	0.8382
	p17	**0.0218**	4.166 (2.000, 45.41)	0.8881	**0.0458**	0.2657
Anaplasma	OmpA	**<0.0001**	28.75 (2.000, 47.05)	**<0.0001**	**<0.0001**	0.0733
	p44	0.7993	0.2250 (2.000, 6911)	0.9616	0.8763	0.9827
	Asp62	**0.0001**	10.61 (2.000, 51.53)	0.6582	**<0.0001**	0.1480
Treponema	TpN17	0.7761	0.2618 (2.000, 42.50)	0.9727	0.9450	0.8963

Supplementary Table S2. Number of patients testing positively (or borderline) for each antigen-specific IgG in individuals hospitalized due to COVID-19 (Group 1), individuals with mild/asymptomatic SARS-CoV-2 infection (Group 2), and individuals not infected with SARS-CoV-2 (Group 3).

**Tested antigens**		**Group 1**	**Group 2**	**Group 3**
Borrelia	VlsE Ba	10	4	1
	VlsE Bg	14	2	2
	VlsE Bs	10	2	0
	p83	7	4	3
	p58	1	2	0
	p41 Ba	20	8	3
	p41 Bs	26	13	5
	p39	2	2	0
	OspB	12	1	0
	OspA Ba	1	4	0
	OspA Bg	2	2	0
	OspA Bs	30	16	6
	OspC Ba	27	13	6
	OspC Bg	21	9	0
	OspC Bs	25	7	0
	OspC Bsp	19	13	5
	NapA	15	10	1
	OspE	0	1	0
	p17	8	6	2
Anaplasma	p44	5	3	3
	OmpA	18	5	0
	Asp62	17	7	3
Treponema	TpN17	1	1	0

Supplementary Table S3. Statistical analysis of *Borrelia*-specific IgM levels in individuals hospitalized due to COVID-19 (Group 1), individuals with mild/asymptomatic SARS-CoV-2 infection (Group 2), and individuals not infected with SARS-CoV-2 (Group 3). Dunnett's T3 multiple comparisons tests were applied; p-values are presented. Antibodies were identified in patients' serum samples with *Borrelia*-dedicated Microblot-Array that quantified IgM levels (U/ml).

Tested antigens		Welch's ANOVA	W(DFn, DFd)	p-values<br>Group 1 v. Group 2	Group 1 v. Group 3	Group 2 v. Group 3
Borrelia	VlsE Ba	0.3696	1.016 (2.000. 47.60)	0.4828	0.8784	0.5515
	VlsE Bg	0.9268	0.07614 (2.000.52.43)	0.9715	0.9982	0.9917
	VlsE Bs	0.4866	0.7331 (2.000. 40.87)	0.9943	0.7770	0.7478
	p83	0.0915	2.505 (2.000. 51.57)	0.1080	0.1257	0.9952
	p58	0.3181	1.179 (2.000. 39.95)	0.6253	0.8126	0.6086
	p41 Ba	0.7616	0.2737 (2.000. 54.11)	0.9997	0.8862	0.9052
	p41 Bs	0.5856	0.5407 (2.000. 52.60)	0.6864	>0.9999	0.7317
	p39	0.7634	0.2715 (2.000. 46.14)	0.9889	0.9758	0.8543
	OspB	0.7055	0.3512 (2.000. 52.78)	0.8564	0.7982	0.9916
	OspA Ba	0.1591	1.908 (2.000. 49.53)	0.1781	0.8981	0.7754
	OspA Bg	**0.0201**	4.254 (2.000. 46.42)	**0.0376**	0.1788	0.3555
	OspA Bs	**0.0003**	9.583 (2.000. 45.25)	**0.003**	**0.0003**	0.7914
	OspC Ba	0.1193	2.212 (2.000. 54.28)	0.4673	0.1131	0.8391
	OspC Bg	0.0514	3.147 (2.000. 51.39)	0.9605	0.0712	0.3635
	OspC Bs	0.0796	2.659 (2.000. 51.28)	0.7362	0.0818	0.6753
	OspC Bsp	**0.03**	3.757 (2.000. 51.76)	0.0679	**0.0281**	0.9983
	NapA	0.0558	3.083 (2.000. 44.19)	0.1997	0.1849	0.9796
	OspE	**0.0148**	4.581 (2.000. 50.91)	**0.0131**	**0.019**	0.9432
	p17	**0.025**	4.006 (2.000. 45.67)	0.0618	0.2128	0.2844
Anaplasma	OmpA	0.9668	0.03378 (2.000. 53.46)	0.9947	0.9999	0.9928
	p44	0.3589	1.046 (2.000. 50.17)	0.3865	0.7766	0.9838
	Asp62	0.9127	0.09152 (2.000. 55.57)	0.9938	0.9627	0.9935
Epstein-Barr virus	VCA-p18	0.1900	1.713 (2.000. 54.23)	>0.9999	0.2576	0.2578

Supplementary Table S4. Number of patients testing positively (or borderline) for each antigen-specific IgM in individuals hospitalized due to COVID-19 (Group 1), individuals with mild/asymptomatic SARS-CoV-2 infection (Group 2), and individuals not infected with SARS-CoV-2 (Group 3).

**Tested antigens**		**Group 1**	**Group 2**	**Group 3**
Borrelia	VlsE Ba	0	1	0
	VlsE Bg	0	0	0
	VlsE Bs	1	1	0
	p83	1	0	1
	p58	1	0	1
	p41 Ba	8	5	1
	p41 Bs	4	5	1
	p39	1	0	2
	OspB	0	0	0
	OspA Ba	0	0	0
	OspA Bg	2	0	0
	OspA Bs	21	5	1
	OspC Ba	18	13	12
	OspC Bg	8	8	4
	OspC Bs	6	4	2
	OspC Bsp	5	2	1
	NapA	0	4	2
	OspE	1	0	0
	p17	1	0	0
Anaplasma	p44	0	1	1
	OmpA	5	0	0
	Asp62	1	1	2
Epstein-Barr virus	VCA-p18	6	4	9

Supplementary Table S5. Multiple logistic regression as a model for testing association between serum levels of IgG antibodies against Osp antigens of *Borrelia* sp. (predictor variable) and hospitalization (outcome variable). Odds ratio, confidence intervals, p-values, and significance are presented; bold- significance with odds ratio>1.0.

Variable	Odds ratio	95% CI	P value	Significance
**OspB**	**1.095**	**1.04 to 1.21**	**0.0096**	**
OspA Ba	0.981	0.960 to 0.994	0.0197	*
OspA Bg	0.9715	0.936 to 0.994	0.0372	*
OspA Bs	0.9945	0.977 to 1.01	0.4621	ns
OspC Ba	0.9862	0.968 to 0.998	0.051	ns
OspC Bg	1.006	0.998 to 1.02	0.1842	ns
**OspC Bs**	**1.03**	**1.01 to 1.07**	**0.0201**	*
**OspC Bsp**	**1.008**	**1.002 to 1.02**	**0.0307**	*
OspE	0.9204	0.834 to 0.981	0.0343	*

Supplementary Table S6. Multiple logistic regression as a model for testing association between serum levels of IgM antibodies against Osp antigens of *Borrelia* sp. (predictor variable) and hospitalization (outcome variable). Odds ratio, confidence intervals, p-values, and significance are presented; bold- significance with odds ratio>1.0.

**Variable**	**Odds ratio**	**95% CI**	**P value**	**Significance**
OspB	0.793	-0.563 to -0.051	0.0552	ns
OspA Ba	0.996	-0.196 to 0.185	0.9615	ns
OspA Bg	1.091	0.002 to 0.235	0.1482	ns
OspA Bs	1.005	-0.00002 to 0.012	0.0861	ns
OspC Ba	1.002	-0.012 to 0.016	0.7991	ns
OspC Bg	0.992	-0.023 to 0.003	0.1651	ns
OspC Bs	0.959	-0.084 to -0.014	0.0136	*
**OspC Bsp**	**1.045**	**0.019 to 0.087**	**0.0065**	**
**OspE**	**1.205**	**0.078 to 0.412**	**0.0141**	*
